# Potential Application of the CRISPR/Cas9 System against Herpesvirus Infections

**DOI:** 10.3390/v10060291

**Published:** 2018-05-29

**Authors:** Yuan-Chuan Chen, Jingxue Sheng, Phong Trang, Fenyong Liu

**Affiliations:** 1 College of Life Sciences and Technology, Jinan University, Guangzhou 510632, China; yuchuan1022@gmail.com; 2 Program in Comparative Biochemistry, University of California, Berkeley, CA 94720, USA; jxsheng@berkeley.edu (J.S.); phong.trang@berkeley.edu (P.T.); 3 National Applied Research Laboratories, Taipei 10636, Taiwan

**Keywords:** CRISPR, clinical application, herpesvirus, latent infection, genome editing, HSV (Herpes Simplex Virus), EBV (Epstein–Barr Virus), CMV (cytomegalovirus), KSHV (Kaposi’s Sarcoma-Associated Herpesvirus)

## Abstract

The CRISPR/Cas9 system has been applied in the genome editing and disruption of latent infections for herpesviruses such as the herpes simplex virus, Epstein–Barr virus, cytomegalovirus, and Kaposi’s sarcoma-associated herpesvirus. CRISPR/Cas9-directed mutagenesis can introduce similar types of mutations to the viral genome as can bacterial artificial chromosome recombination engineering, which maintains and reconstitutes the viral genome successfully. The cleavage mediated by CRISPR/Cas9 enables the manipulation of disease-associated viral strains with unprecedented efficiency and precision. Additionally, current therapies for herpesvirus productive and latent infections are limited in efficacy and cannot eradicate viruses. CRISPR/Cas9 is potentially adapted for antiviral treatment by specifically targeting viral genomes during latent infections. This review, which focuses on recently published progress, suggests that the CRISPR/Cas9 system is not only a useful tool for basic virology research, but also a promising strategy for the control and prevention of herpesvirus latent infections.

## 1. Introduction

Diseases caused by viruses are difficult to treat due to the high viral mutation rate and latent infections. Particularly, it is almost impossible to eradicate latent viruses in the human host. In this review, we focus on the therapy of herpesviruses such as herpes simplex virus 1 (HSV-1), herpes simplex virus 2 (HSV-2), Epstein–Barr virus (EBV), cytomegalovirus (CMV), and Kaposi’s sarcoma-associated herpesvirus (KSHV) [1]. The clustered regularly interspaced short palindromic repeats (CRISPR)/Cas9 nuclease system [2,3], a genome editing technology, represents a novel and exciting possibility for the treatment of herpesvirus infections in recent studies [4]. Here, we briefly describe the characteristics of herpesviruses and the CRISPR/Cas9 system.

### 1.1. Properties of Herpesviruses

Herpesviridae, also known as Herpesviruses, is a large family of DNA viruses that cause human diseases and remain an important global health problem [1]. Herpesviruses can cause lytic or latent infections, in which the viruses either enter a lytic pathway to lyse their host cells or a latent pathway to stay silent within the host cells [5,6,7,8]. The productive form of herpesviruses is a spherical enveloped virion with a diameter of 120 to 260 nm (usually about 150 nm). It consists of a large double-stranded, linear DNA genome encoding 100–200 genes wrapped in an icosahedral capsid, surrounded by amorphous protein called the tegument, with an outer lipid bilayer membrane envelope containing different glycoproteins. Herpesviruses replicate in their host’s nucleus, with a sequential transcription and translation of immediate–early (IE), early (E), and late (L) genes, with the earlier genes regulating the transcription of later genes. HSV-1, HSV-2, EBV, human CMV (HCMV), and KSHV are distinct viruses in this family known to cause diseases in humans [5,6,7,8] (Table 1).

### 1.2. Life Cycle of Herpesviruses

The life cycle of herpesviruses can be classified into lytic and latent infection stages. Infection is initiated when an infectious virion attaches to a host cell with specific receptors on the cell surface. Following binding of the viral envelope glycoproteins to cell membrane receptors, the virion penetrates the host cell through receptor-mediated endocytosis or membrane fusion. The viral capsid breaks down to release the viral DNA genome. The invading viral DNA takes over the host cell and manipulates its enzymes to produce new viral DNA genomes and proteins. The new viral DNA genome and proteins assemble to form new virions. Finally, new virions break free to search for new host cells. During symptomatic infections, mRNAs are expressed from viral lytic genes in infected cells. However, during latent infections, a small number of viral genes may transcribe latency-associated transcripts (*LAT*) in some host cells. In this fashion, viruses can persist in host cells indefinitely. Though primary infection may be accompanied by limited clinical illness, long-term latency has no signs and symptoms.

### 1.3. Latency and Reactivation of Herpesviruses

Virus latency is the ability of a virus to lie dormant within its hosts, denoted as the lysogenic part of the viral life cycle [1]. A latent infection is a type of persistent viral infection, where the virus is still present in the host and causes no overt symptoms but can be transmitted to others. When viruses are stimulated by stress or when their host immune system is suppressed, the dormant virus can reactivate to start producing large amounts of viral progeny to cause symptoms and illness. There are two kinds of viral latency: proviral latency and episomal latency. For proviral latency, a provirus integrates into the host genome. Human immunodeficiency virus (HIV) is a great example of establishing proviral latency. HIV enters the host genome as a provirus, and its proviral DNA replicates synchronously with the host. Episomal latency does not require the integration of host and viral genome and refers to the use of genetic episomes during latency. The herpesvirus family utilizes episomal latency to maintain its viral genome. Herpesvirus viral DNAs can reside in the cytoplasm or nucleus as linear or lariat structures. Compared with a proviral genome, episomal viral DNAs are more vulnerable to cellular sensors or degradation by the host’s enzymes. Both proviral and episomal latency may require maintenance for continued infections and fidelity of viral genes. Latency is generally maintained by viral genes expressed primarily during latency, in which the *LAT* genes’ products can keep the viral genome from being destroyed. Reactivation of latent viruses has been implicated in many diseases. When herpesviruses are reactivated, the transcription of viral genes switches from *LAT* genes to lytic genes to enhance viral DNA replication and virion production. Clinically, the reactivation to lytic infections is often accompanied by nonspecific symptoms such as low-grade fever, headache, sore throat, malaise, and rash [1]. In addition, this reactivation may lead to death in some serious cases.

### 1.4. The Clustered Regularly Interspaced Short Palindromic Repeats (CRISPR)/Cas9 Nuclease System

**Figure 1:**
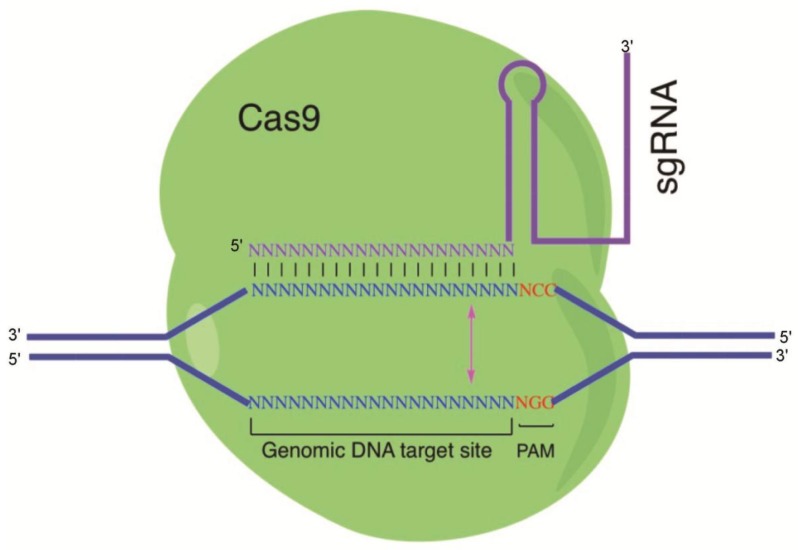
Illustration of the CRISPR/Cas9 system: The Cas9 protein interacts with the single-guide RNA (sgRNA) to direct endonuclease activity proximal to the protospacer adjacent motif (PAM) sequence. Custom-designed sgRNAs recognize their target sequence and allow Cas9 endonuclease to cleave the sense strand 3 base pairs (bp) and antisense strand 3 bp upstream of the PAM sequence (NGG). Binding of sgRNAs to the target sites induces Cas9 endonuclease to create a double-strand break (blunt end) on the genomic target.

The CRISPR/Cas system originated from a prokaryotic immune system that confers resistance to foreign genetic elements such as plasmids and viruses (bacteriophages) and provides an acquired immunity for the host [2,3]. CRISPRs are loci that contain multiple, short, direct repeats of DNA sequences. Each repeat contains a series of base pairs followed by about 30 base pairs known as “spacer DNA”. The spacers are short segments of DNA from a virus and serve as a ‘memory’ of past exposures [2,3]. When encountering this specific phage again, the host can recognize the foreign DNAs by complementation with the stored short spacer sequence in CRISPR RNA (crRNA). Hybridization between crRNA and the complimentary foreign sequence initiates destruction of the invading DNA/RNA by Cas nucleases. Back in 2012, researchers adopted CRISPR/Cas9 in the mammalian system. Since then, CRISPR application in mammalian systems has been a tremendous success. Cas9 is an element of the CRISPR/Cas system in *Streptococcus pyogenes*. As part of *Streptococcus pyogenes*’ adaptive immunity process, foreign DNA is acquired as a spacer in the CRISPR locus. crRNA is matured by pre-crRNA annealing with transactivating RNA (tracrRNA) followed by RNase III cleavage. The mature crRNA and tracrRNA form a complex with Cas9 protein to cleave the target DNA [9]. For easier application in gene editing, scientists developed plasmids that could express crRNA and tracrRNA together as single-guide RNA (sgRNA). sgRNA along with Cas9 protein can edit any spot in the genome as long as the target sequence is followed by an NGG protospacer adjacent motif (PAM) sequence. Cas9 is a DNA endonuclease whose structure is bilobed, composed of target recognition domain and nuclease lobes. The nuclease lobe contains nucleases RuvC, HNH, and a carboxyl-terminal domain for the PAM recognition [10] (Figure 1). When sgRNA and Cas9 are expressed in the mammalian system, Cas9 protein can bind to the host genome PAM sequence through 3D diffusion [11,12]. The GG dinucleotide is recognized through major-groove interactions with conserved arginine residues from the C-terminal domain of the Cas9 protein. Interactions of the minor groove of the PAM duplex and the phosphodiester group at the +1 position in the target DNA strand lead to local strand separation immediately upstream of the PAM [13]. If the target DNA matches the sgRNA sequence, the Cas9 HNH endonuclease domain cleaves the complementary strand, and the Cas9 RuvC cleaves the noncomplementary strand domain at 3 nucleotides (nt) upstream of the PAM [14]. Studies have shown that nonspecific target binding events are transient and short-lived compared with the specific target binding event. Once Cas9/sgRNA binds to its specific target, the Cas9 protein goes through conformational changes to accommodate target DNA cleavage [11]. The double-stranded break (DSB) created by Cas9 can be repaired by either Non-Homologous End Joining (NHEJ) or Homologous Repair. Homologous repair can happen when donor homologous DNA is present. Otherwise, NHEJ will repair the DSB. NHEJ will introduce a small insertion/deletion (indel) in the targeted DNA and will create in-frame amino acid deletions, insertions, or frameshift mutations. This leads to premature stop codons within the open reading frame (ORF).

## 2. CRISPR/Cas9 as a Tool for Studying Herpesvirus Host Interaction

The CRISPR/Cas9 system has been used in prokaryotic and eukaryotic cells for genome editing such as silencing, enhancing, or modification of specific genes [2,3]. By constructing plasmids containing Cas9 genes and a specifically designed sgRNA, the organism’s genome can be cleaved at most locations with the only limitation being the availability of the PAM binding sequence, NGG, in the targeting site. CRISPR/Cas9 was first shown to work as a genome engineering or editing tool in human cell culture, animals, and bacteria [2]. Efficient genome modifications have been performed in species such as baker’s yeasts (*Saccharomycetes cerevisiae*) [15], zebrafish [16], nematodes (*Caenorhabditis elegans*) [17], plants [18], and mice [19]. Additionally, the CRISPR/Cas9 system has been successfully applied in basic research for herpesviruses by engineering, targeting, activating, or repressing specific genes of interest [4,20]. These findings and their implications may be discussed in the broadest context possible. Future research directions may also be highlighted.

### 2.1. HSV

The human interferon-inducible factor 16 (*IFI16*) is an antiviral factor that binds nuclear viral DNA and promotes antiviral responses [21]. It is a nuclear protein that is involved in the regulation of transcription and induction of interferon-β (*IFN-β*), and in the activation of inflammation. To investigate the role of *IFI16* in controlling HSV infection, CRISPR/Cas9 technology was used to generate knockouts of *IFI16* [21,22]. These studies showed that *IFI16* represses HSV-1 gene expression to reduce virus titers by acting as a restriction factor for HSV-1 and preventing the association of important transcriptional activators with wild-type HSV-1 promoters. It restricts wild-type HSV-1 replication and may play a direct or indirect role in histone modification [21,22].

UL7, an HSV-1 tegument protein, is highly conserved in viral infections and proliferation, though its mechanism of action is still poorly understood. The replication rate of the HSV-1 *UL7* mutant (*UL7*-MU strain) was 10-fold lower than that of the wild-type (WT) strain [23]. The pathologic effect of the *UL7*-MU strain was attenuated in infected mice compared with the wild-type HSV-1 strain. In latency, the expression of viral *LAT* in the central nervous system and trigeminal nerve was lower in *UL7*-MU-infected mice than that in mice infected with wild-type HSV-1. By modulating the transcription of the immediate early gene *α4*, UL7 may be involved in transcriptional regulation through interacting with the transcript complex structure of the viral genome during HSV-1 infection [23]. In the study showing that the mutated tegument protein UL7 attenuated the virulence of HSV-1, the *UL7*-MU strain was constructed using CRISPR/Cas9 technology [23].

### 2.2. EBV

EBV is etiologically responsible for Burkitt’s lymphoma and several human cancers, in which viral genomes are maintained as multicopy episomes [6]. EBV infection in gastric epithelial cells triggers malignant transformation by inducing resistance to oncogene-induced cell death. It is extremely difficult to clone viral DNA because EBV-positive cancer cells are incompetent for progeny virus production in cell culture. By CRISPR/Cas9-mediated strand break of the viral genome, bacterial artificial chromosome (BAC) clones of EBV episomes were obtained [24]. EBV strains maintained in two gastric cancer cell lines (SNU719 and YCCEL1) were cloned, and their complete genome sequences were determined [24]. EBV variant strains may also be relevant to EBV-associated diseases, and the determination of their viral genome sequences will facilitate the identification of any disease-specific EBV strains. CRISPR/Cas9-mediated cleavage of EBV episomal DNA was found to enable the cloning of disease-associated viral strains with unprecedented efficiency and precision [24]. As two gastric cancer cell-derived EBV strains were cloned, the infection of epithelial cells with reconstituted viruses revealed the mechanism of EBV-mediated epithelial carcinogenesis. The relationship between viral genome variation and EBV-associated diseases should be established by these experiments [24].

### 2.3. CMV

Human CMV (HCMV) only propagates inside human cells; thus, it has developed methods to protect itself against host stress responses and take over cellular processes to complete its life cycle [7]. The mammalian target of rapamycin complex 1 (mTORC1) controls cell growth and anabolic metabolism, and functions as a critical host factor activated by HCMV during successful infection [25]. Since mTORC1 is crucial for virus replication, HCMV maintains high mTORC1 activity. mTORC1 inhibitors suppressed HCMV replication *in vitro* and reduced the incidence of HCMV reactivation in transplant recipients [25]. The multifunctional HCMV protein pUL38 can activate mTORC1 by binding and antagonizing tuberous sclerosis complex protein 2 (TSC2) and plays a major role in blocking endoplasmic reticulum stress-induced cell death during HCMV infection [25]. Therefore, pUL38 inhibitors are a potential antiviral factor for HCMV infection and a mutant host cell line is needed. In the study showing that pUL38 can activate mTORC1 both through TSC2-dependent and -independent manners, the *TSC2* knockout U373-MG strain was created using CRISPR/Cas9 technology [25].

The CMV genome is complex and its adaptations to cell culture have complicated the study of infection *in vivo* [7]. Recombination engineering of CMV BACs enabled the study of mutant CMVs. CRISPR/Cas9-based mutagenesis has been used to construct mutants of guinea pig CMV (GPCMV), which can be used to infect guinea pigs, an animal model, to study CMV-associated pathogenesis and diseases [26]. An alternative strategy for the mutagenesis of guinea pig CMV that utilizes CRISPR/Cas9-mediated genome editing can introduce the same type of target mutations to the viral genome as BAC-based methods. This method is highly efficient in introducing targeted insertions or deletions to nonessential viral genes. Viral mutants can be recovered after limited viral replications, minimizing the risk of spontaneous mutations. CRISPR/Cas9 avoided selection that might occur by BAC recombination engineering and facilitated genetic manipulation of low-passage or clinical CMV isolates [26].

### 2.4. KSHV

KSHV, a human oncogenic virus, adapts specific mechanisms to manipulate its host cellular microenvironment and hijacks the host signaling pathways to maintain latent infections [5]. The pathogenesis of KSHV is closely related to its modulation of cellular signaling pathways, including the extracellular signal-regulated kinase (ERK) pathway and the mitogen-activated protein kinase (MAPK) pathway [20]. KSHV protein ORF45 activates the cellular kinase RSK (p90 ribosomal S6 kinase, a major functional mediator of ERK/MAPK signaling), and this activation is vital for KSHV gene expression and virion production. Also, ORF45 is shown to contribute to the sustained activation of both ERK and RSK during KSHV lytic replication [20]. The difference in protein phosphorylation is significant upon induction of KSHV lytic replication in that the activation of RSK by ORF45 causes differential phosphorylation of cellular and viral substrates [20]. A phosphoproteomic analysis of KSHV-infected cells was performed to characterize the specific substrates of ORF45-activated RSK [27]. *RSK* was knocked out by CRISPR/Cas9; additionally, several cellular substrates were identified by screening and the consequent effects on the regulation of gene expression and virion production were characterized [27]. In the study to implicate ORF45-mediated activation of *RSK* in the regulation of viral and host gene expression during KSHV lytic replication, the *ORF45/RSK*-dependent phosphorylation was validated by CRISPR/Cas9-mediated knockout of *RSK* in KSHV-infected cells [27].

## 3. Treatment of Herpesvirus-Associated Diseases Based on CRISPR/Cas9

The advances in genome editing using CRISPR/Cas9 promote virological studies and may provide a cure for persistent herpesvirus infections by directly targeting these viruses within infected cells. Herpesvirus infection is currently treated using nucleoside analogs such as Ganciclovir, Valganciclovir, and Foscarnet [1], but these drugs are designed to block viral polymerase activity [7]. As the primary course of treatment is using nucleotide analogues to block viral polymerase replication, another difficulty in herpesvirus infection treatment is the failure to control reactivation of the viruses. The treatment is only effective when the virus genome is actively replicating; hence, the remaining silenced viral genome can be reactivated at any time. With that being said, it is crucial to develop other treatments against herpesvirus infections. We need to develop a treatment strategy that could disrupt the viral genome. To conclude, a gene therapy that can edit and eliminate the herpesvirus genome is the only solution for curing herpesvirus latent infections. Here, we review recent applications of the CRISPR/Cas9 system and discuss its therapeutic potential to treat the productive and latent infections of herpesviruses [20,28,29,30].

Roehm et al. adapted the CRISPR system to treating HSV-1 infection [31]. Roehm and colleagues designed sgRNAs targeting *ICP0*, a crucial viral-encoded protein that can regulate viral gene expression and replication. Although sgRNA targeting *ICP0*, an immediate early gene that promotes transcription from the viral gene, exhibited a significant decrease in viral production, a mixture of sgRNAs targeting *ICP0*, *ICP4*, and *ICP27* eliminated HSV viral infection. These promising results suggest CRISPR as a possible solution for treating HSV infection.

In 2016, Diemen et al. conducted a study that used CRISPR/Cas9 technology to suppress herpesvirus virus replication in both latent and lytic infection models [4]. This study showed CRISPR’s ability to suppress three prototypical members of the herpesvirus family: EBV, HSV, and HCMV. For EBV, the authors designed sgRNAs for both the latent infection model and the lytic infection model. For the latent infection model, sgRNAs were designed to target EBV viral miRNAs *miR-BART5*, *miR-BART6*, and *miR-BART16*. The EBV latently infected gastric carcinoma cells were transduced to express CRISPR/Cas9 sgRNAs. A luciferase assay followed by sequencing showed that the targeted miRNAs were downregulated and edited. This experiment illustrates the application of CRISPR technology in a herpesvirus latent infection model. For the lytic infection model, the authors designed sgRNAs targeting the viral EBV nuclear antigen 1 (EBNA1) and several areas within the EBV origin of replication (OriP). EBV-GFP infected cells had an almost complete loss of GFP after a combination of sgRNAs treatment. A similar approach was tested in both HCMV and HSV. Both lytic infection models showed almost complete suppression of viral infections. Additionally, CRISPR gene editing could abrogate replication of HSV-1 reactivated from quiescence.

Other groups also proved the effectiveness of CRISPR/Cas9 in treating latent herpesvirus infections. Wang and Quake investigated the application of CRISPR/Cas9 in treating latent virus infection in EBV [32]. They designed several sgRNAs that targeted the EBV genome location responsible for the viral structure, transformation, and latency. Patient-derived cells with latent Epstein–Barr virus infection were treated with the designed CRISPR/Cas9. The results showed the elimination of EBV genome in a quarter of the cells, and half of the cells showed a decrease in viral load after CRISPR/Cas9 treatment. Yuen et al. designed sgRNAs targeting EBV genome region *EBNA1*, *OriP*, and *W repeats* [33]. Their study showed a reduction of 50% in viral genome copy number. Although the suppression of the EBV viral genome did not affect EBV latently infected cells, the remaining infected cells were sensitized to chemotherapy.

While it is difficult to find good animal models for human herpesvirus infections, there are several studies providing proof of concept evidence of CRISPR/Cas9 technology treating viral infections in the animal model. Lin et al. conducted a study using CRISPR gene editing technology to treat HBV infection both *in vitro* and *in vivo* [34]. They first tested the efficacy of sgRNAs targeting HBV viral genomes *in vitro*. Then, the top two sgRNAs that had highest targeting efficiency were selected for application in an *in vivo* study. They used the well-established HBV hydrodynamics-mouse model of HBV infection. HBV expression vector and the CRISPR/Cas9 dual expression vectors were coinjected into the tail veins of C57BL/6 mice by hydrodynamic transfection. Serum HBsAg levels were significantly reduced in mice receiving HBV-specific sgRNAs targeting the HBV viral genome. Sequencing results showed 27.8% of the clones contained indel in the targeted region. In another study, Yin et al. demonstrated the CRISPR/Cas9 system in excising the HIV-1 provirus in different animal models representing the different levels of clinical relevance [35]. They intravenously injected quadruplex sgRNAs/saCas9 AAV-DJ/8 into mice. The results showed that CRISPR/Cas9 treatment excised HIV-1 proviral DNA and significantly reduced viral RNA expression in several organs/tissues of mice. Additionally, successful proviral excision was detected by PCR genotyping in different organs. Both studies reviewed above showed the ability of delivering a CRISPR/Cas9 system effectively and safely to the targeted cells and organs.

Taken together, this progress in adapting CRISPR/Cas9 in suppressing herpes viral replication, and the advances in delivering the CRISPR system in animal models provide hope for finding a cure for herpesvirus infections.

## 4. Challenges of CRISPR/Cas9 Delivery

Delivery tools for transfection or gene transfer are agents to facilitate nucleic acids entering target cells. Their main function is to increase the transfection efficiency of DNA (including genes, plasmid DNA, and DNA fragments) and RNA (including mRNA, miRNA, and siRNA) *in vitro* or *in vivo*. The most common strategies for CRISPR/Cas9 delivery are lipoids [36,37], viruses [36,37], nanoparticles [36,37], bacteria [38], gene guns [39], electroporation [40], and nanostraws [41] (Table 2).

CRISPR/Cas components must be transported to the target cells to exert a therapeutic effect. The delivery will be critical to the success of therapeutic genome editing applications. Therefore, the delivery tool is essential for CRISPR/Cas9 delivery to target cells and it is very crucial to select a suitable delivery tool with high specificity, efficiency, and safety. However, the options of CRISPR/Cas9 delivery tools present challenges due to the following issues.

1. The delivery tool is not specific enough: Some delivery tools are not very specific and may deliver nucleic acids to nontarget cells. It is important to reduce the risk of nonspecific delivery, but the evaluation of their benefits and risks is complex.
2. The delivery tool is not very efficient: Not all delivery tools are efficient; some of them are low in efficiency and require multiple rounds of transfections. Additionally, it is hard to improve and evaluate their efficiency, especially in animals and clinics.
3. The delivery tool is deficient in biosafety: Some delivery tools are toxic, biohazardous, or even destructive to normal cells or recipient hosts. Some delivery tools such as lipoids, viruses, bacteria, and nanoparticles may induce vector-associated immune responses in hosts, and immune barriers must be overcome [36]. Thus, verifying their safety in preliminary testing is needed.

In several recent studies, encouraging progress has been made to possibly overcome the challenges of delivering CRISPR/Cas9 *in vivo* (Table 3). Adeno-associated viruses (AAV) with low immunogenicity enter the cells by endocytosis upon binding to the specific integrin and receptor and integrate at a specific site called AAVS1 in the host genome. This site-specific integration avoids unpredictable insertion mutation and other harmful consequences [42]. A novel approach integrates large single-strand transgene cassettes into the genomes, increasing the knock-in efficiency by combining CRISPR/Cas9 with AAV by 13.6–19.5-fold compared with conventional AAV-mediated methods [43]. A single administration of lipid nanoparticles (LNP) that are biodegradable and well tolerated can deliver CRISPR/Cas9 components to achieve high-efficiency *in vivo* genome editing with a concomitant reduction of the mouse transthyretin (TTR) gene serum protein [44]. The packaging of the newly discovered smaller Cas9 and its guide RNA into one AAV delivery vehicle allows for efficient *in vivo* genome editing. The combination of small Cas9 orthologues, tissue-specific promoters, specific AAV serotypes, and different routes of delivery has improved the efficiency and precision for *in vivo* application and overcome the immunogenicity and toxicity of the delivery tools [45].

## 5. Conclusions

CRISPR/Cas9, a naturally occurring component of a bacterial immune system, uses a novel nuclease system to protect bacteria from bacteriophage infections and has been harnessed for a variety of genome editing applications. By delivering the Cas9 protein and appropriate guide RNAs into a cell, the organism’s genome can be cut at most locations with the availability of the PAM binding sequence. It can also be modified to make programmable transcription factors that allow scientists to target and activate or repress specific genes. Several studies have illustrated the utility of CRISPR/Cas9 technology for the study of herpesviruses including HSV, EBV, CMV, and KSHV. Specific genes and factors/proteins have proved to be essential for herpesvirus proliferation and latency through CRISPR/Cas9-mediated engineering in host or viral genomes. These promising results in basic research contribute to an increased understanding of herpesviruses, the establishment of specific cell lines or potential animal models, as well as the development of new drugs for viral infections.

The pharmaceutical studies have made great achievements in drug discovery, and therapeutic options have been expanded to include small molecules (e.g., siRNAs), antiviral agents, protease inhibitors, and preventive vaccines for diseases caused by viral infections [1]. However, effective treatments for persistent, recurrent, and highly prevalent herpesviruses are still unavailable. This represents a significant unmet medical need for herpesvirus infections such as HSV, EBV, HCMV, and KSHV. Current antiviral therapy is not able to eradicate latent viruses in humans efficaciously, though the standard drugs are effective for the treatment of lytic infection and alleviation of symptoms in patients. The advance of CRISPR/Cas9 technology presents a novel and promising strategy to treat latent herpesvirus infections. Previous work has shown that CRISPR/Cas9 can be adapted for antiviral treatment for latency *ex vivo* and/or *in vivo*. Furthermore, there is encouraging evidence that the ever-important delivery challenge can be overcome with the use of AAV delivery vectors and lipid nanoparticles. CRISPR/Cas9 may have the potential to be an effective therapy against latent viral infections in humans, though more preclinical studies will be needed to demonstrate its effectiveness *in vivo*.

## Figures and Tables

**Table 1:** Major human herpesviruses (HHV-1, -2, -4, -5, and -8).

Type (Synonym)	Subfamily	Primary Target Cells	Latency Cells	Pathophysiology
HHV-1 (HSV-1)	α (alpha)	Mucoepithelial cells	Sensory neurons	Oral or genital herpes (predominantly orofacial), cold sores, keratitis, etc.
HHV-2 (HSV-2)	α (alpha)	Mucoepithelial cells	Sensory neurons	Oral or genital herpes (predominantly genital), etc.
HHV-4 (EBV)	γ (gamma)	B cells, Epithelial cells	B cells, Epithelial cells	Infectious mononucleosis, Burkitt’s lymphoma, nasopharyngeal carcinoma, Hodgkin’s disease, post-transplant lymphomas, CNS lymphoma in AIDS patients, etc.
HHV-5 (HCMV)	β (beta)	Monocytes, Lymphocytes, Epithelial cells	Peripheral monocytes, CD34+ progenitor cells	Infectious mononucleosis-like syndrome, retinitis, pneumonitis, gastrointestinal diseases, mental retardation, vascular disorders, etc.
HHV-8 (KSHV)	γ	Lymphocytes and other cells	B cells, Mononucleocytes	Kaposi’s sarcoma, primary effusion lymphoma, some types of multicentric Castleman’s disease, etc.

HSV-1: herpes simplex virus 1; HSV-2: herpes simplex virus 2; EBV: Epstein–Barr virus; HCMV: human cytomegalovirus; KSHV: Kaposi’s sarcoma-associated herpesvirus; CNS: central nervous system; AIDS: acquired immunodeficiency syndrome.

**Table 2:** Delivery tools for CRISPR/Cas9.

Delivery Tools	Example	Characteristic
Lipoid	Lipofectamine, Liposome	The lipid subunits which form liposomes entrap the transfection materials, allowing themselves to overcome the electrostatic repulsion of the cell membrane to let DNA or RNA cross into the cytoplasm to access the nuclei or organelles.
Virus	Lentivirus, Adenovirus, Adeno-associated virus (AAV), Baculovirus	A specific virus is engineered to deliver DNA or RNA to target cells and used as a vector for gene transfer.
Nanoparticle	Mesoporous silica nanoparticles (MSNs), Dendrimers, Carbon Nanotubes, Cationic polymers	Nanoparticles (1–100 nanometers in size), consist of a variety of compounds and materials, can be complexed with DNA or RNA for gene delivery.
Bacterium	*Salmonella*	An attenuated strain of *Salmonella* which is invasive but nonpathogenic shows DNA transfer activity with little cytotoxicity and pathogenicity in hosts.
Gene gun	PDS-1000/He Particle Delivery System	The device, a biolistic particle delivery system, is used for delivering exogenous DNA to cells; the payload is an elemental particle of a heavy metal coated with DNA.
Electroporation	Electroporator	An electric field is applied to cells to increase the cell permeability, allowing DNA to be introduced into the cell.
Nanostraw	Navan	The device is used for creating a direct physical conduit to cells for DNA delivery, mimicking the gap junction between cells.

**Table 3:** Possible strategies for overcoming the challenges for CRISPR/Cas9 delivery.

Challenge	Strategy
Specificity	Discovery of a specific virus such as adeno-associated viruses (AAV).
Efficiency	Application of a combination system such as AAV-CRISPR.
Biosafety	Combination with several factors such as smaller Cas9 orthologues, tissue-specific minimal promoters, AAV serotypes, and different routes of administration; Development of novel and safe delivery tools such as lipid nanoparticles (LNP), AAV, and baculoviruses.
